# Inpatient Rehabilitation: Prediction of Changes in Sensorimotor Performance in Multiple Sclerosis: A Pilot Study

**DOI:** 10.3390/jcm10102177

**Published:** 2021-05-18

**Authors:** Philipp Gulde, Joachim Hermsdörfer, Peter Rieckmann

**Affiliations:** 1 Center for Clinical Neuroplasticity Medical Park Loipl, Medical Park SE, Thanngasse 15, 83483 Bischofswiesen, Germany; p.rieckmann@medicalpark.de; 2 Department for Sports and Health Sciences, Technical University of Munich, 80992 Munich, Germany; joachim.hermsdoerfer@tum.de

**Keywords:** multiple sclerosis, rehabilitation, sensorimotor, prediction, Watzmann Severity Scale

## Abstract

Inpatient rehabilitation has been shown to be an effective intervention for sensorimotor performance in multiple sclerosis (MS) patients. So far, predictions of the rehabilitation outcomes are limited. The objective was to predict inpatient rehabilitation outcomes by changes in the Watzmann Severity Scale (WSS), a statistical estimation of the EDSS by sensorimotor capacity. Sensorimotor performance and physical activity during rehabilitation (by actigraphy) were assessed in a sample of 28 MS patients at a facility for neurorehabilitation. Daily changes in the WSS were predicted by a model of multiple linear regression. The resulting model had an R^2^_adjusted_ of 0.48 (*p* < 0.01) and revealed five impacting factors (a reduction in the WSS represents an improvement): the number of steps (β-weight = 0.52, *p* < 0.01), the duration of nocturnal rest time (β-weight = 0.46, *p* = 0.01), the EDSS at entry (β-weight = 0.38, *p* = 0.03), a relapsing-remitting MS (β-weight = 0.37, *p* = 0.03), and the performance in a visuomotor pursuit task with time pressure (β-weight = −0.35, *p* = 0.04). One standard deviation improvement was predicted when the patient at admission yielded 6600 fewer steps per day, 94 min less rest per night, −2.7 points in the EDSS at entry, a relapsing-remitting MS, and a pursuit task performance that decreased by 2.2 standard deviations. Overall, the patients improved by −0.22 ± 0.51 WSS points during 19.3 ± 4.5 d of inpatient rehabilitation. Different potential explanations of the findings are discussed, one of which proposes that the results reflect an unhealthy lifestyle which, in addition to MS, would explain the higher predicted improvements by rehabilitation tackling both MS and the patients’ lifestyle.

## 1. Introduction

Although inpatient rehabilitation is used frequently as a measure to improve function in multiple sclerosis (MS) patients, there is only little objective data on its success. In a recent study, we introduced the Watzmann Severity Scale (WSS) [1], a sensorimotor based statistical estimation of the Expanded Disability Status Scale (EDSS) [2], in order to sensitively examine the changes during inpatient rehabilitation in MS patients. The underlying idea was to have a sensitive surrogate of the EDSS, so that changes due to a rehabilitation intervention could be displayed based on a well-known and widely used clinical scale. By the use of the WSS, we anticipated that we would be able to show even subtle changes in sensorimotor performance over the course of the rehabilitation. The nine-hole peg test (as part of the Multiple Sclerosis Functional Composite [3]), with reported minimal detectable changes of 19.4% (dominant upper-limb) and 29.1% (non-dominant upper limb) [4], is in line with a coarsely estimated necessary difference of 20.5% of the summed trial durations of the dominant and the non-dominant hand to achieve statistical significance from our previous study [1]. In comparison, other tests were revealed to be much more sensitive (e.g., finger tapping with a detected change of 4%). However, the assessed parameters were only able to predict 4% of the variance of changes in the WSS and the duration of the rehabilitation was not a significant factor in the model in our prior study [1]. In 1999, Langdorn and Thompson [5] published a study explaining 57% of the variance of the motor scale of the Functional Independence Measure (FIM) [6] due to neurorehabilitation (3 weeks of multidisciplinary therapy at a neurorehabilitation facility; 97% of the sample were in the progressive phase of the disease). They identified three main factors, which were the verbal intelligence assessed by the Wechsler Adult Intelligence Scale-Revised Vocabulary [7] (positive impact), the FIM motor scale on admission (negative impact), and the cerebellar function score, which is a subset of the EDSS (negative impact of impairment). Interestingly, although in an extremely weak model, the only factor in our prediction model was a visuomotor control task with high demands on temporal resolution (a worse performance predicted worse rehabilitation outcomes [1]). This is in line with Langdorn’s findings on cerebellar function. Unfortunately, Langdorn and Thompson did not report beta-weights, but according to their regression method, it can be seen—and it was discussed by the authors—that the main influencing factor was the FIM on admission. This however introduces a potential bias, since sources for improvements for lower functioning patients are manifold, while a ceiling effect can be reached in patients with higher functional capacities. Cattaneo et al. [8] focused on the effects of in- and outpatient rehabilitation on balance by the Berg Balance Scale [9]. The authors reported higher odd ratios to positively respond to rehabilitation in patients with a higher balance disability (a potential similar effect as described by Langdorn and Thompson [5]). Grasso et al. [10] examined the effects of inpatient rehabilitation on functional outcomes (i.e., Barthel Index [11] and Rivermead Mobility Index [12]) and found that the improvements were lower in patients with higher EDSS grades (7.0–8.5). For both functional measures, they identified the pyramidal score of the EDSS as a negative predictive factor with models explaining 7.7% of the variance of changes in the Barthel Index and 1.6% of the variance of changes in the Rivermead Mobility Index.

Overall, there is very little known about the prognostic factors for the effectiveness of inpatient rehabilitation in MS. Many of the studies used measures that were susceptible to bias and the overall outcomes were only able to predict a minimal part of the variance (except Langdorn and Thompson [5]). So far, it appears that rehabilitation is most effective in patients with a lower disability [1,5,10] (excluding the FIM ceiling bias in [5]). In order to further examine potential predictive factors such as physical activity levels, we included behavioral data by actigraphy into our study. By this, we wanted to gather data on daytime behavior (metabolic rate, steps, and fatigability) and nocturnal rest patterns in addition to objective, quantitative parameters from tests of sensorimotor function, and patient characteristics.

We hypothesized that in addition to a lower disability status at admission, patients with lower physical activity would show higher improvements in the WSS. We decided to use the WSS, since it is a model of the EDSS and, at the same time, it offers a more sensitive estimation of changes (basically the EDSS with “higher resolution”), as we were able to show in Gulde et al. [1]. However, the WSS is not the same as the EDSS and changes in sensorimotor performance due to rehabilitation would result in a performance that would be expected of a person with a certain EDSS. Therefore, it remains an estimate that does not necessarily display clinical changes. Nevertheless, we expected to gather information on factors that could influence the effect of a rehabilitation intervention. This study used the WSS as a tool but did not aim to provide proof of predictive validity, since we did not expect the effects of rehabilitation that can be displayed by the EDSS. Future studies should test this by examining the course of progression over several years with different tools including the WSS, EDSS, and others such as the Multiple Sclerosis Functional Composite. It is important to note that actigraphy is usually unable to reliably detect, or validly depict, certain training contents such as strength training or balance exercises. Therefore, we hypothesized the negative association between physical activity and improvements in the WSS, since we anticipated that patients that walk and move more in addition to their physical therapy were not challenged enough by their inpatient schedule (and would therefore show less of an improvement due to rehabilitation).

## 2. Materials and Methods

### 2.1. Sample

We assessed a total of 28 patients (46% male, 54% female), with a mean age of 46.3 ± 11.2 a (23–73 a). The mean EDSS of patients was 3.3 ± 1.4 (1.0–6.5, median: 3.5), the WSS was 3.36 ± 1.00 (1.66–5.77, median: 3.22). A total of 68% of cases were categorized as relapsing-remitting and 32% were categorized as primary or secondary progressive MS. The mean duration since the patient reported the first manifestation of neurological symptoms was 13.9 ± 11.7 a (0–46 a). The patients were recruited at the Center for Clinical Neuroplasticity Medical Park Loipl (Medical Park SE) in Bischofswiesen, Germany. All patients gave written informed consent. Ethical approval was given by the ethics committee of the Medical Faculty of Technical University of Munich.

### 2.2. Procedure

The sensorimotor performance was assessed twice with a mean interval of 19.0 ± 4.1 d (10–25 d). In each session, patients’ WSS was assessed (a model including grip strength, finger tapping frequency, postural sway, and smoothness of gait, see: Gulde et al. [1]) in addition to a set of further sensorimotor tests. Part of those tests was conducted using a smartphone (Microsoft Lumia550, Microsoft Corporation, Redmond, USA; programmed via Microsoft Visual Studio 2017, Microsoft Corporation, Redmond, USA). Additionally, patients wore an acceleration sensor (ActiGraph wGT3X-BT, ActiGraph LLC, Pensacola, USA, 100 Hz measurement frequency downsampled to 1 Hz) for one full week (including weekends) on their dominant wrist (the least impaired wrist). For analyses, the Freedson algorithms were used [13]. The first author of the study (single rater) conducted all the assessments. The first session was close to the entry (within the first week) at the rehabilitation facility and the second session was close to the release.

### 2.3. Parameters

We assessed a set of sensorimotor parameters, which included the Watzmann Severity Scale (WSS) as an estimation of the EDSS [1]. The WSS comprises of grip strength, finger tapping frequency, postural sway, and smoothness of gait: WSS = −3.688 × COMPLEXITY − 0.219 × TAP − 0.060 × SWAY − 0.013 × GRIP + 9.894. Higher values correspond with higher levels of disability (as in the EDSS). Further, the average daily changes of the WSS were calculated (DELTA), with improved sensorimotor performance resulting in negative values. The upper-limb performance was assessed firstly by the summed grip strength (GRIP) in kg (as the mass equivalent to force on earth) using a hand-dynamometer (Deyard, EH101, Deyard Ltd., Shenzhen, China). Secondly, the upper-limb performance was assessed by the summed finger tapping frequency (TAP) measured in Hz over 10 s (the use of the whole upper-limb was allowed) via smartphone; tapping has been associated with cortical excitability and motor conductivity [14]). Thirdly, the upper-limb performance was measured by the sum of the performance in a visuomotor pursuit task with increasing spatio-temporal demands over the course of 20 s (PURSUIT) in mm (assessed via smartphone at 30 Hz measurement frequency; distance to a pseudo-randomly moving and steadily accelerating target). Finally, the upper-limb performance was measured by the sum of the trial durations to perform the nine-hole peg test (PEG) in s to assess fine motor control. All sums of tests were based on the performance of the dominant and non-dominant upper-limb. Further, the mean simple reaction time to a visual stimulus (RT) over 10 trials in ms was assessed as a measure of reaction speed (influenced by arousal, wakefulness, and processing speed).

The performance of the lower-limbs and the postural system was tested firstly by the (inversed) sway of the frontal waistline during a parallel stance over 10 s (SWAY) in s^2^/m (assessed via smartphone; 1000 divided by the mean rectified acceleration at 20 Hz measurement frequency). Secondly, the performance of the lower-limbs and the postural system was tested by the maximum gait velocity, i.e. the time to walk 10 m (WALK) in s, and the smoothness of gait during normal paced walking over 10 s (COMPLEXITY). Smoothness was assessed via smartphone. The relation of the arc length and the integral of a frequency spectrum of the acceleration signal (20 Hz) at the sternum was used as a measure of smoothness and energetic economy: the ratio is set in relation to a single frequency signal and ranges from 0% (worst performance) to 100% (best performance). By the combination of maximum walking speed and smoothness of gait, we aimed to assess both the physical gait capacity and the adapted sensorimotor skills.

Actigraphy was used over one full week (starting at the first session) to gather a coarse estimate of physical activity by the number of steps (STEPS) and the energetic activity by the body mass adjusted metabolic equivalent (MET); steps could be taken running or slowly walking, so this added information to the number of steps. Indicators of fatigability by the ratio of the quantity of longer (≥10 min) and shorter (≥5 min) activity bouts (RATIO) [15], and rest and sleep behavior by the average duration of nocturnal rest (absence of activity during nighttime) (REST) in h (nocturnal rest as absence of activity (>60 min) during nighttime (>18:00)) were also used to gather coarse estimates of physical activity.

Patient characteristics that were included into the analysis were the type of MS (TYPE), being either relapsing-remitting (= 0) or progressive state (= 1). Further, the biological sex of the participant (SEX) (male = 0, female = 1), the age (AGE) in a, the estimated clinical severity of MS measured by the Expanded Disability Status Scale (EDSS) (additional to the WSS), the time since the first patient-reported manifestation of neurological symptoms as an estimate of the disease duration (MANIFEST), an estimate of the body composition by the body mass index (BMI) in kg/m^2^ (none of the participants was an active body builder or pro sport), and the time between assessments in days as the duration of the observed rehabilitation program (DAY) in d, which was not ultimately the length of the stay at the facility, was noted. Further, we included whether the patient was currently receiving a disease modifying therapy (DMT) (no = 0, yes = 1) and the number of present comorbidities (COM), defined as diagnosed depression (29%), elevated blood pressure (11%), orthopedic indications (32%), diabetes mellitus (7%), restless leg syndrome (7%), post-traumatic stress disorder (7%) and so on. Due to the heterogeneity of present comorbidities, we were not able to include subanalyses for, for instance, different orthopedic indications.

### 2.4. Statistical Analysis

The necessary sample size was determined using G*Power (version 3.1.9.7, University of Düsseldorf, Düsseldorf, Germany) [16]: two-sided a priori for linear multiple regressions with α = 0.05 and a power of 0.80. We estimated an explained variance of 44% (based on Langdorn and Thompson [5], excluding the Wechsler Adult Intelligence Scale Vocabulary) and 19 potential predictors, resulting in a necessary sample size of 26.

A model of multiple linear regression was computed for DELTA by sensorimotor (only the first session) and behavioral parameters as well as patient characteristics. The critical α for statistical significance was set to 0.05. The threshold for the variance inflation factor (VIF) was set to 5.0. The statistical analysis was performed with RStudio (RStudio Inc., Boston, USA).

## 3. Results

### 3.1. Descriptives

**Figure 1:**
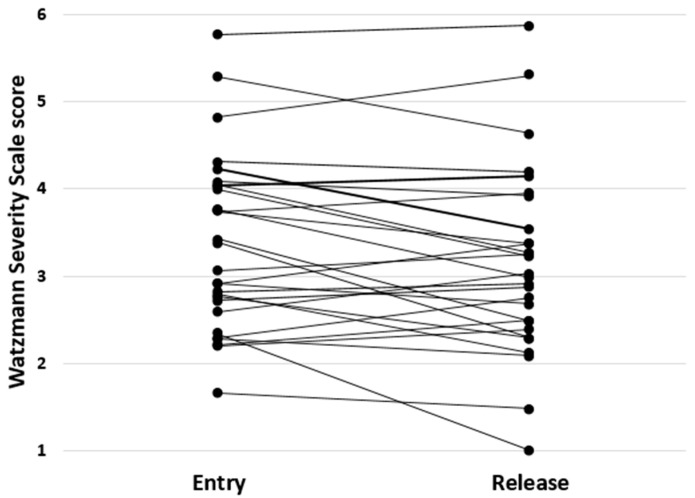
WSS scores at the entry and the release of all 28 patients. Each line represents the change of the WSS of a single patient over the course of inpatient rehabilitation.

On average, the patients improved by −0.22 ± 0.51 (−1.33–0.48) points on the WSS over 19.3 ± 4.5 d of inpatient rehabilitation (Figure 1). The daily improvement on the WSS (DELTA) was −0.011 ± 0.028 (−0.065–0.034). Patient characteristics, sensorimotor performance, and the results of actigraphy are given in Table 1.

### 3.2. Prediction of DELTA

**Figure 2:**
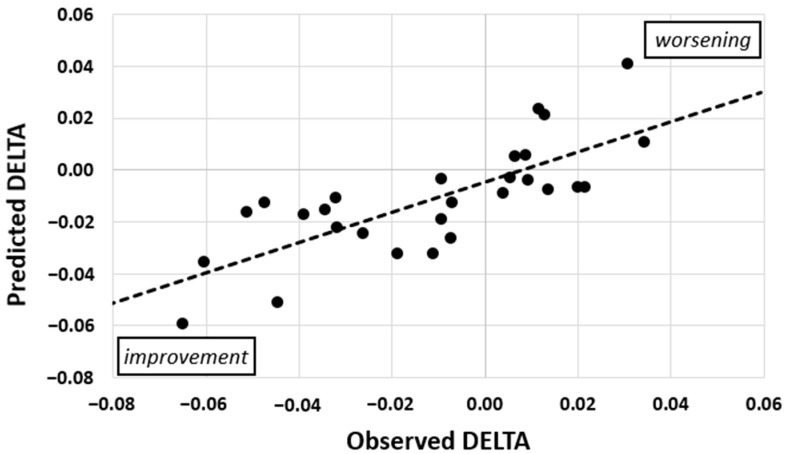
Predicted and observed daily changes in the WSS (DELTA) of 28 patients. The model had an R^2^_adjusted_ of 0.48 (*p* < 0.01) and five factors. Values below zero represent an improvement of the sensorimotor function over the course of rehabilitation.

The model of multiple linear regression for DELTA had an R^2^_adjusted_ of 0.48 (unadjusted R^2^ = 0.58, *p* < 0.01, Figure 2, Table 2) and five predictors: STEPS, REST, EDSS, TYPE, and PURSUIT. To give an illustration, we calculated the necessary difference for one standard deviation improvement of the predicted values by the factors. This resulted in 6600 fewer steps walked per day, a 2.7 points lower EDSS score at entry, 2.5 mm lower accuracy per hand in the pursuit task or a performance that was reduced by 2.2 standard deviations, 94 min less inactivity per night, and a relapsing-remitting form of MS (1.01 standard deviations in DELTA prediction).

## 4. Discussion

In this pilot study, we predicted the changes in the WSS of 28 MS patients during inpatient rehabilitation. Patients revealed a total improvement of −0.22 in the WSS score (which is similar to our prior results [1]). We hypothesized that, in addition to a lower disability at entry, which was suggested by the literature [1,5,10] (excluding the FIM bias in [5]), lower levels of physical activity assessed by actigraphy would predict higher improvements. A model of multiple linear regression was strong with 48% explained variance (*p* < 0.01) and revealed five impacting factors: the number of steps, nocturnal rest time, the EDSS at entry, the type of MS, and the performance in a visuomotor pursuit task with time pressure. A patient that was walking less and resting less during the night, with a lower EDSS, a relapsing-remitting MS, and a worse performance in the pursuit task would have a better predicted rehabilitation outcome. This might seem counterintuitive at a first glance. Grasso [10] and Langdorn and Thompson [5] suggested that patients with too high a disability might have severe problems participating in physical therapy. This would have suggested that grip strength, the nine hole peg test, the 10 m walk test, or the postural sway to be good predictors. However, they showed no significant impact. In addition, higher step quantities and a better performance in the pursuit task predicted less of an improvement. Still, the impact of lower EDSS grades remains.

If one takes an unhealthy lifestyle into consideration, this could potentially include fewer steps (actigraphy included one weekend), less nocturnal rest, and worse coordinative abilities (less trained by daily activities), the absence of a daily reminder of a neurological threat (that would be found in higher disability levels and a progressive form; “physical capacity is good enough to procrastinate action”), and a higher tendency to rely on disease modifying therapy (preventing relapses instead of counteracting a progressive disability). The association between nocturnal rest and the ratio of activity bouts (R^2^ = 0.23, *p* < 0.01, less sleep was associated with higher ratios) supports this hypothesis; less sleep leads to increasing levels of fatigability or, vice versa, increased levels of fatigability also impairs sleep. It is important to note that the BMI of patients and patients currently receiving a disease modifying therapy were not included in the model. The missing impact of DMT could be explained by its strong redundancy with the disease type; only 16% of relapsing-remitting patients received no DMT, while 67% of progressive forms did not receive a DMT. This unhealthy lifestyle hypothesis would be one potential, and highly speculative explanation, that assumes one common underlying factor (which would not result in such low VIFs (Table 2)). However, some research groups have observed an interaction between the lifestyle and the disability controlled/adjusted physical health of MS patients [17] and diet, severe fatigue, depression, pain, or cognitive impairment [18], as well as an interaction between experiences of fluctuations of symptom severity and lifestyle [19], indicating a potentially relatively fast reactivity of symptomatology to lifestyle/dietary changes. Taken together, in patients with MS and an unhealthy lifestyle, rehabilitation could counteract both and could therefore show stronger improvements. However, the missing significant impact of BMI, DMT, and the number of comorbidities goes against this hypothesis and indicates that it should be further tested with an emphasis on lifestyle factors. In this case, it might also be recommended to include the biological sex or gender into such analyses, since it has been shown that men and women show significantly different lifestyle patterns concerning relevant health factors [20], especially in physical activity, which is lower for adolescent females [21].

Alternatively, additional steps could originate from patients not being challenged and exhausted enough by their therapy measures, and therefore adding steps to their daily schedules (as an additional factor to weekend and leisure time activities). In agreement with the literature, lower EDSS grades would allow for greater participation in physical therapy, the same as an extended daily activity window (and therefore less nocturnal rest). A relapsing-remitting form of MS can stand for more focal damage in the central nervous system (an earlier disease state (i.e., less accumulated disability) was excluded by a missing variance inflation of EDSS and TYPE, and age was not significant) resulting in greater potential of cortical plasticity and allowing for greater neural repair of damage [22] (although age was not a predictor). A reduced pursuit performance could suggest a ceiling effect on the other end (the best performers) of the spectrum (the distribution of PURSUIT was skewed to the left), a similar bias as in the study by Langdorn and Thompson [5]. An examination of PURSUIT at release showed that there was no significant change in skew (*p* = 0.31; 0.08 at entry to 0.14 at release), which contradicts the assumption of a ceiling effect (both sides of the performance distribution were able to improve/deteriorate). It is important to note that the performance in the pursuit task is not part of the WSS model. In this sense, the predictive value of PURSUIT is in the indication of a patient characteristic (in terms of a coordinative capacity) that is either moderating changes in the WSS due to rehabilitation or is a symptom of a certain behavioral pattern (i.e., either MS impacted the performance and this physiological state is influencing or enabling the rehabilitation outcome or the performance is a result of a behavioral pattern that is either fostering or attenuating the capacity to execute the task).

One noteworthy limitation of the study was the limited sample size. A post-hoc analysis using G*Power [16] resulted in an achieved power of 0.794 (R^2^_unadjusted_ = 0.578, 5 predictors), so our results and interpretation should be followed with a certain caution; confirmative studies seem warranted at this point. Further, we decided to use the WSS instead of the EDSS due to its increased sensitivity, while at the same time estimating this widely used clinical measure (the EDSS). Other candidates for such assessments such as the Multiple Sclerosis Functional Composite revealed weaker associations with the EDSS [23] compared to the WSS. However, one should be aware that the WSS is a statistical estimate of the EDSS and is not able to display certain features such as reduced bladder control, impaired swallowing, or dementia. Although we were able to observe comparable changes to our prior study [1], it must be kept in mind that we were not directly assessing changes in myelination, which could have been tested by central motor conduction time [24,25,26] (with Armutlu et al. [24] not being able to show changes in central motor conduction time due to rehabilitation measures).

## 5. Conclusions

In conclusion, the statistical model that predicts 48% of variance in sensorimotor performance changes during inpatient rehabilitation raises a significant number of questions that should be further pursued in order to better understand the potential of patients visiting rehabilitation facilities and therefore to maximize the effect of such measures. However, the improvement of −0.22 WSS points within an average of less than three weeks is already a significant improvement of sensorimotor function and further studies examining the sustainability of such interventions seem warranted.

## Figures and Tables

**Table 1:** Patient characteristics, sensorimotor performance, and actigraphy of 28 MS patients.

Parameter	Mean, STD, and Range	Parameter	Mean, STD, and Range	Parameter	Mean, STD, and Range
Age in a	46.3 ± 11.2 23–73	Grip in kg	68.0 ± 19.2 33.3–97.2	Steps	93.4k ± 31.4k 44.6k–183.5k
EDSS	3.3 ± 1.4 1.0–6.5	Tap in Hz	12.1 ± 1.9 7.4–16.0	Met	1.42 ± 0.10 1.26–1.61
Manifest in a	13.9 ± 11.7 0–46	Sway in s^2^/m	11.5 ± 3.9 0.9–18.9	Ratio	6.5 ± 3.7 2.4–15.0
Sex	46% male 54% female	RT in ms	479 ± 50 387–583	Rest in h	7.4 ± 1.0 5.2–9.4
Type	68% rel. rem. 32% progr.	Pursuit in mm	13.7 ± 2.3 10.1–20.1	DMT	68% yes 32% no
BMI in kg/m^2^	25.9 ± 4.5 18.2–35.1	Complexity	62 ± 20% 29–92%	COM	1.2 ± 1.3 0–5
Day in d	19.3 ± 4.5 10–29	Walk in s	7.9 ± 3.9 4.4–24.1		
WSS_entry_	3.4 ± 1.0 1.7–5.8	PEG in s	49.9 ± 13.9 33.2–92.0		
WSS_release_	3.1 ± 1.1 1.0–5.9				
DELTA	−0.011 ± 0.028 −0.065–0.034				

EDSS: expanded disability status scale, Manifest: Time since first patient-reported manifestation of symptoms, rel. rem.: relapsing remitting, progr.: primary or secondary progressive, BMI: body mass index, WSS: Watzmann severity scale, Grip: summed grip strength, Tap: summed finger tapping frequency, Sway: inversed body sway, RT: simple reaction time, Pursuit: summed distance in a pursuit task, Complexity: smoothness index of gait, Walk: time to cover 10 m, PEG: nine hole peg test, Steps: number of steps over a full week, Met: body mass adjusted metabolic equivalent, Ratio: ratio between the quantity of 5 min and 10 min activity bouts, Rest: nocturnal rest, DMT: disease modifying therapy, COM: quantity of comorbidities.

**Table 2:** Model of multiple linear regression for DELTA with an R^2^_adjusted_ of 0.48 (*p* < 0.01).

	STEPS	REST	EDSS	TYPE	PURSUIT
β-weight	0.52	0.46	0.38	0.37	−0.35
VIF	1.27	1.50	1.32	1.28	1.33
*p*	<0.01	0.01	0.03	0.03	0.04

Positive β-weights correspond to less improvement by higher values, e.g., more steps predict higher DELTAs and therefore less improvements during inpatient rehabilitation.

## Data Availability

Data is available on request.
